# Does Root Development Status Affect the Outcome of Tooth Autotransplantation? A Systematic Review and Meta-Analysis

**DOI:** 10.3390/ma15093379

**Published:** 2022-05-08

**Authors:** Ernest Lucas-Taulé, Anna Bofarull-Ballús, Marc Llaquet, Montse Mercade, Federico Hernández-Alfaro, Jordi Gargallo-Albiol

**Affiliations:** 1 Department of Oral and Maxillofacial Surgery, Universitat Internacional de Catalunya, C/Josep Trueta s/n St Cugat del Vallés, 08195 Barcelona, Spain; bofarullanna@uic.es (A.B.-B.); h.alfaro@uic.es (F.H.-A.); jgargallo@uic.es (J.G.-A.); 2 Private Practice, Dental Esthetic Bcn, C/ Francesc Carbonell, n 21., 08034 Barcelona, Spain; mllaquet@uic.es; 3 Biomedical Research Institute (IDIBELL), 08908 Barcelona, Spain; montsemercade@ub.edug; 4 Department of Dentistry, Universitat de Barcelona, 08907 Barcelona, Spain

**Keywords:** closed apex, complete root formation, incomplete root formation, meta-analysis, open apex, systematic review, tooth autotransplantation

## Abstract

**Background:** Tooth autotransplantation is defined as the surgical repositioning of an autogenous tooth in another surgical site within the same individual. **Aim:** The aim of this research was to analyze the outcome of tooth transplantation using immature donor teeth compared with closed apex teeth and to compare differences between donor tooth positions on the arch. **Methods:** Electronic and manual literature searches were performed in different databases, including the National Library of Medicine (MEDLINE), EMBASE (OVID), Cochrane Central (CENTRAL), and the digital library of the Universitat Internacional de Catalunya (UIC University) from 1978 to March 2021. Studies were selected when they fulfilled the following criteria: only human prospective clinical studies, minimum sample size of 10 patients, minimum follow-up of 1 year, studies reporting or with at least deducible data on survival rates, immediate tooth autotransplantation with completed or incomplete root formation, and publications in the English language. A meta-analysis of random effects was developed to estimate the global effect measure of the survival rate, success rate, and root resorption involving the total sample, as well as open- and closed-apex groups. **Results:** Twenty-four articles were eligible for analysis. The Cohen’s kappa corresponding to this review was 0.87, and the risk assessment was considered low–moderate for the included studies. Overall survival and success rates were 95.9% and 89.4%, respectively, with a mean follow-up of 4 years and an overall mean age of 25.2 ± 12.3 years. Closed apex teeth showed a survival rate of 3.9% lower than that of open apex teeth. Higher complication rates were found for both inflammatory external root resorption and replacement root resorption in the closed-apex group, without reaching statistical significance. **Conclusions:** Tooth autotransplantation is a viable treatment alternative, regardless of the apical condition, with high survival and success rates after a mean follow-up of 4 years. Open-apex donor teeth could be considered the gold-standard option, showing lower complication rates when compared to closed-apex donor teeth. Future randomized controlled clinical studies are needed to examine the long-term prognosis of this technique.

## 1. Introduction

Tooth autotransplantation is the surgical repositioning of an autogenous tooth in another surgical site within the same individual. The main indications for this procedure include congenitally missing teeth or those involving ectopic eruption and tooth loss due to traumatic injuries, caries, or periodontal disease [1]. Although osseointegrated implants for tooth replacement have high long-term survival rates, they are not free from complications [2,3,4]. The prevalence of peri-implant diseases has been reported in several studies, ranging from 19 to 65%, showing a clear positive correlation between function time and prevalence of peri-implant pathology [4,5,6]. Therefore tooth autotransplantation should be considered in young patients as a treatment option, as it might help delay implant placement [7,8].

Given the osteogenic potential of the periodontal ligament (PDL) cells attached in the donor tooth, bone formation can be expected at the recipient site, as long as PDL cells remain preserved during tooth transplantation [8,9]. Thus, the surgical procedure requires gentle manipulation of the donor tooth [10] and reduced extraoral time [1], ensuring successful PDL healing [10,11,12].

Since Slagsvold and Bjercke established a tooth autotransplantation protocol at the University of Oslo in the 1960s, the predictability of this treatment has been supported by several long-term follow-up studies [9,13,14,15]. Czochrowska et al. reported a survival rate of 90% after an observation period of 17–41 years [9]. A similar survival rate (97.5%) was observed by Jonsson et al. after up to 22 years (mean observation period 10 years) [15]. Survival predictors of the transplanted tooth chiefly include preservation of the donor tooth PDL cells [1,10].

However, the success rates for tooth autotransplantation range from 0% [16] to 100% [11], depending on the observation period and factors related to the donor tooth [17], patient, and surgical procedures [18]. The lack of a clear consensus on the success criteria for this treatment has resulted in contrasting findings across studies [19].

A comprehensive assessment and understanding of all the prognostic factors influencing the outcome of tooth autotransplantation is important to achieve success with this surgical technique. Therefore, the aims of this systematic review and meta-analysis were (I) to analyze the overall outcome of tooth transplantation in young patients with incomplete root formation compared with adult patients with complete root formation; and (II) to compare the success rates of tooth transplantation between different donor tooth positions on the arch.

## 2. Materials and Methods

This systematic review protocol was registered in the PROSPERO International Prospective Register of Systematic Reviews hosted by the National Institute for Health Research, University of York, Centre for Reviews and Dissemination (code number CRD42020180854).

### 2.1. Patient, Intervention, Comparison, Outcome (PICO) Question

This review was conducted according to the preferred reporting items for systematic reviews and meta-analysis (PRISMA) guidelines [20] and the patient, intervention, comparison, outcomes (PICO) design: patients underwent immediate tooth autotransplantation to replace a missing tooth (P), considering tooth autotransplantation performed in young patients involving incomplete root formation (I), compared to tooth autotransplantation in adults involving complete root formation (C). Survival rate was the primary outcome, and success rate, periodontal condition (inflammatory root resorption and replacement root resorption), pulp condition (pulp obliteration and pulp healing), and root formation (arrested, completed, or incomplete) were the secondary outcomes (O). Thus, the focused question of this systematic review was, “Does the root development of the donor teeth affect the prognosis and the clinical outcomes of tooth autotransplantation?”.

### 2.2. Eligibility Criteria

Studies were selected if they fulfilled the following criteria: (1) only human prospective clinical studies; (2) minimum sample size of 10 patients; (3) minimum follow-up of 1 year; (4) studies reporting or at least with deducible data on survival rates; (5) immediate tooth autotransplantation involving a definitive tooth with complete or incomplete root formation; and (6) publications in English. We excluded studies involving cryopreservation or intentional replantation.

### 2.3. Information Sources and Search Strategy

Electronic and manual literature searches were performed by two independent authors (E.L.T. and A.B.B.), covering the period from 1978 to March 2021, in databases including the National Library of Medicine (MEDLINE), EMBASE (OVID), Cochrane Central (CENTRAL), and the digital library of the Universitat Internacional de Catalunya (UIC University). Recent systematic reviews and meta-analyses related to tooth autotransplantation were also screened, and some authors were contacted to obtain further information and clarify some reported data.

The search strategy combined different terms: ((((((tooth autotransplantation[Title] OR ((“tooth”[MeSH Terms] OR “tooth”[All Fields] OR “teeth”[All Fields]) AND (“transplantation, autologous”[MeSH Terms] OR (“transplantation”[All Fields] AND “autologous”[All Fields]) OR “autologous transplantation”[All Fields] OR “autotransplantation”[All Fields]))) OR ((“transplantation”[MeSH Terms] OR “transplantation”[All Fields] OR “transplanted”[All Fields]) AND (“tooth”[MeSH Terms] OR “tooth”[All Fields] OR “teeth”[All Fields]))) OR ((“transplantation”[MeSH Terms] OR “transplantation”[All Fields] OR “transplanted”[All Fields]) AND (“tooth”[MeSH Terms] OR “tooth”[All Fields]))) OR ((“tooth”[MeSH Terms] OR “tooth”[All Fields]) AND (“transplantation”[Subheading] OR “transplantation”[All Fields] OR “transplantation”[MeSH Terms]))) OR ((“tooth”[MeSH Terms] OR “tooth”[All Fields] OR “teeth”[All Fields]) AND (“transplantation”[Subheading] OR “transplantation”[All Fields] OR “transplantation”[MeSH Terms]))) OR ((“tooth”[MeSH Terms] OR “tooth”[All Fields]) AND autotransplanted[All Fields])) OR ((“tooth”[MeSH Terms] OR “tooth”[All Fields] OR “teeth”[All Fields]) AND autotransplanted[All Fields]). Additionally, a manual search was conducted until March 2021 in dental journals, including the *Journal of Clinical Periodontology*, the *Journal of Endodontics*, the *International Endodontic Journal*, the *Journal of Periodontology*, the *International Journal of Oral and Maxillofacial Surgery*, the *American Journal of Orthodontics and Dentofacial Orthopedics*, and the *European Journal of Orthodontics*.

### 2.4. Selection of Studies

Two independent reviewers (E.L.T. and A.B.B.) selected and examined all titles and determined which abstracts to evaluate. All duplicate investigations were removed, selected abstracts were screened for possible inclusion, and publications were identified for full-text analysis. The reviewers obtained the full texts of the selected studies. Finally, a third reviewer (J.G.A.) analyzed the studies in accordance with the inclusion criteria to confirm the selected studies, which were analyzed individually. The Cohen’s kappa corresponding to this review was 0.87.

### 2.5. Data Extraction

The following information was extracted from each article: (1) author and year of publication; (2) patient sample; (3) test and control group characteristics (open or closed apex); (4) survival rate; (5) success rate; (6) periodontal condition, classified into inflammatory external root resorption (IER) and replacement root resorption (RRR); (7) pulp condition (pulp obliteration and pulp healing); and (8) root formation, classified as arrested, completed, or incomplete. An arrested condition is defined as the absence of root development or changes in the root length after an immature tooth autotransplantation. All data were independently extracted by two reviewers (E.L.T. and A.B.B.). Some studies reported ambiguous information regarding study design; therefore, some authors were contacted for further clarification in order to validate whether their investigations met our inclusion criteria. Any disagreement between the investigators was resolved by a third reviewer (J.G.A.).

### 2.6. Quality Assessment

The criteria used to evaluate the quality of the selected prospective studies were according to ROBINS-I, a tool for assessing risk of bias in non-randomized studies of interventions [21]. Two independent reviewers (E.L.T. and A.B.B.) evaluated the quality of the selected studies, and any disagreement was resolved by a third author (J.G.A.). The studies were evaluated for three components according to ROBINS-I: pre-intervention, at intervention, and post-intervention. 

### 2.7. Statistical Analysis

Statistical analysis was performed using R 3.5.1 (R Core Team, R Foundation for Statistical Computing, Vienna, Austria). A meta-analysis of random effects was developed to estimate the global effect measure of the survival rate, the success rate, and the root resorption involving the total of the sample, the open-apex group, and the closed-apex group. Forest charts were used to visualize the results with 95% confidence intervals (CI), and the I^2^ index of heterogeneity was also calculated.

A meta-regression model with a moderating variable of the apex type and under the random effects approach was estimated to compare all the studies. The influence of the position of the autotransplanted tooth was also evaluated. A 5% level of significance was used in all analyses (α = 0.05).

## 3. Results

### 3.1. Study Selection

**Figure 1:**
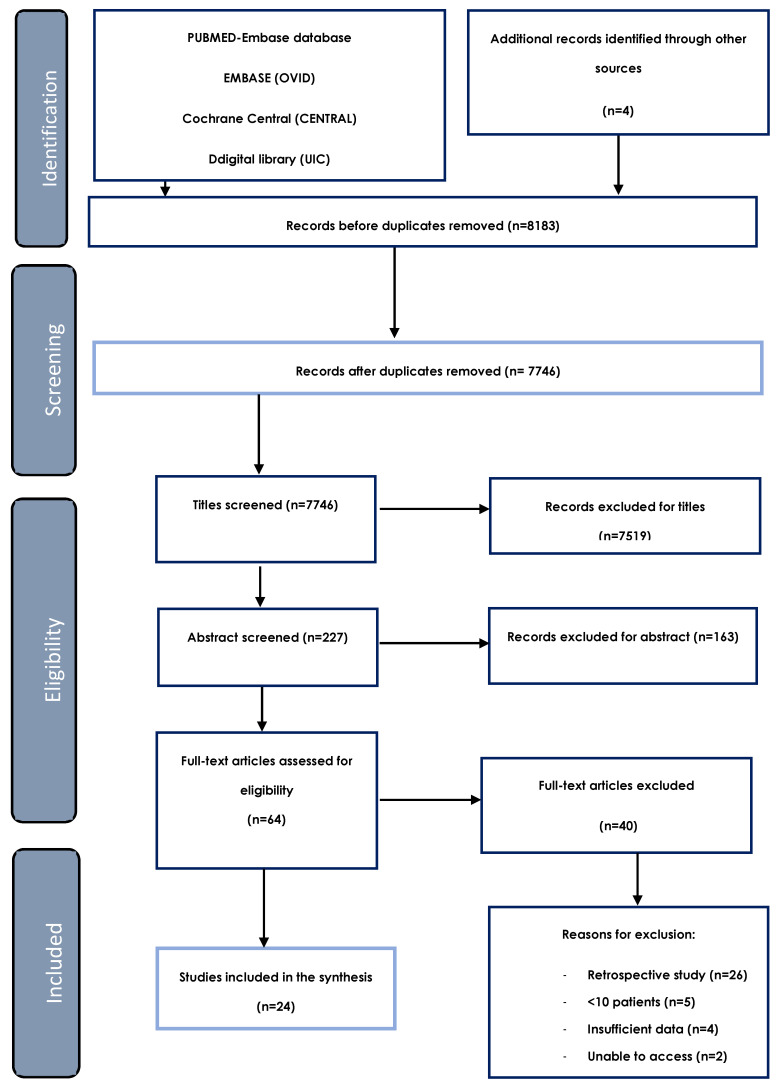
PRISMA flow chart of the screening process in the different databases.

The initial electronic search resulted in 8179 articles, of which four were retrieved by manual search. After screening the titles and abstracts, 64 articles qualified for full-text review. Forty were excluded for lacking sufficient data to answer the objectives proposed or because the research methodology did not fulfill the inclusion criteria. Ultimately, 24 articles were considered eligible for qualitative and quantitative analyses (Figure 1).

### 3.2. Study Characteristics

All 24 studies corresponded to prospective case series studies, treating a total of 1516 autotransplanted teeth: 12 studies with 987 teeth in the open-apex group) [10,11,12,22,23,24,25,26,27,28,29,30,31,32,33] and 10 studies with 453 teeth in the closed-apex group [10,12,18,22,27,29,34,35,36,37,38,39], for a total of 22 studies and 1440 autotransplanted teeth. Two studies [40,41] (76 autotransplanted teeth) did not define the apex condition but were included in the present analysis. The total follow-up period was 4.07 ± 2.17 years, and the overall mean age was 25.2 ± 12.3 years. The open-apex and closed-apex groups had a mean follow-up period of 3.46 ± 1.62 and 5.67 ± 2.12 years, respectively, and a mean age of 15.7 ± 4.6 and 36.9 ± 8.3, respectively (Table 1).

### 3.3. Quality Assessment

Fifteen studies had moderate risk of bias quality scores, whereas nine had a low risk of bias score according to ROBINS-I for prospective case series studies (Table 2).

### 3.4. Survival Rate

**Figure 2:**
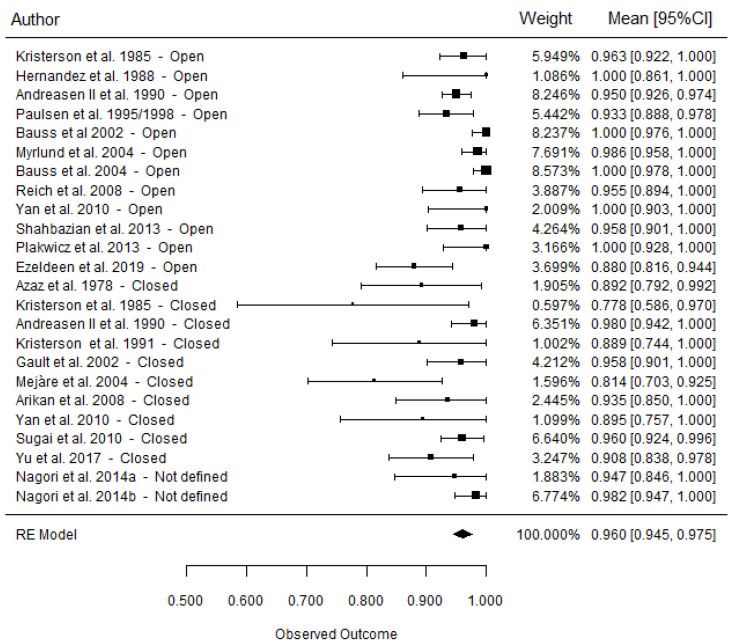
Forest plots for the overall survival rate (mean [95% CI]).

**Figure 3:**
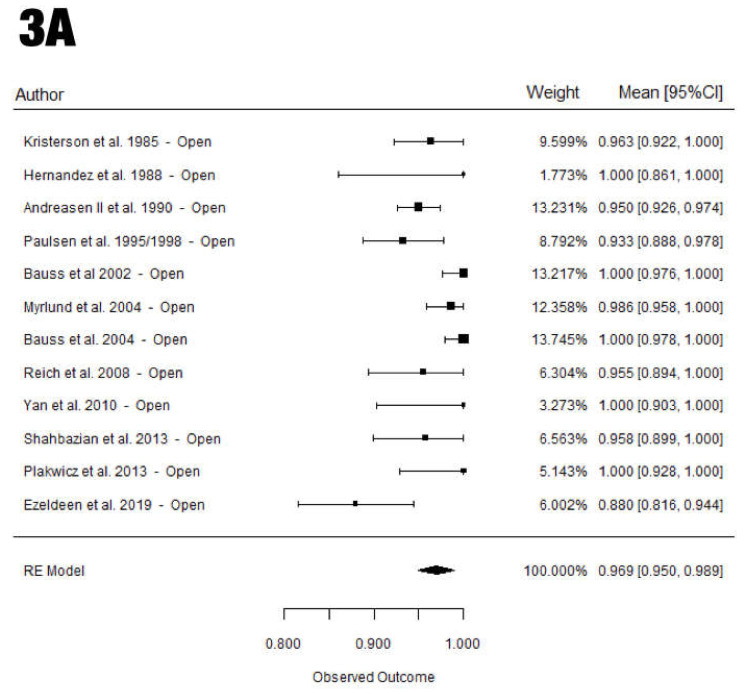

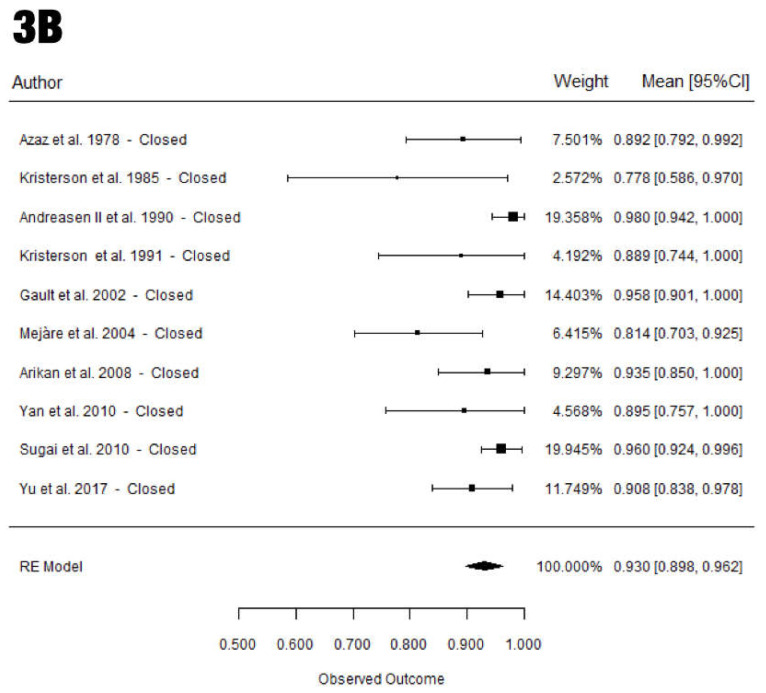
Forest plots for the open-apex (**A**) and closed-apex (**B**) overall survival rate (mean [95% CI]). (**A**) Open-apex survival rate. (**B**) Closed-apex survival rate.

An overall survival rate of 95.9 ± 0.8% was reported (Figure 2), with a moderate heterogeneity for all studies (I^2^ = 52.7%). The average survival rate in the open-apex group was 96.9 ± 1.0%, which was associated with a high heterogeneity for all included studies (I^2^ = 61.8%) compared to the closed-apex group (93.0 ± 1.7%), which showed moderate heterogeneity (I^2^ = 46.1%). The closed-apex group had a survival rate 3.9% lower than that of the open-apex group, but the difference was not significant (*p* = 0.052) (Figure 3).

In the open-apex group, the premolars and third molars showed a survival rate of 95.5 ± 1.2% and 99.7 ± 0.8%, respectively, both groups reaching statistical significance (*p* = 0.008). However, in the closed-apex group, no significant differences were observed when comparing canines, premolars, and third molars (*p* = 0.137), which showed 91.6 ± 3.3%, 90.2 ± 9.8%, and 88.4 ± 2.6% mean survival rates, respectively (Table 3 and 4).

Non-statistical differences in the survival rate of premolars were observed according to the type of apex (*p* = 0.797). In contrast, significant differences were observed in the third-molar group, with the closed-apex group showing a significantly lower survival rate than that of the open-apex group (*p* < 0.001) (Table 4).

### 3.5. Success Rate

**Figure 4:**
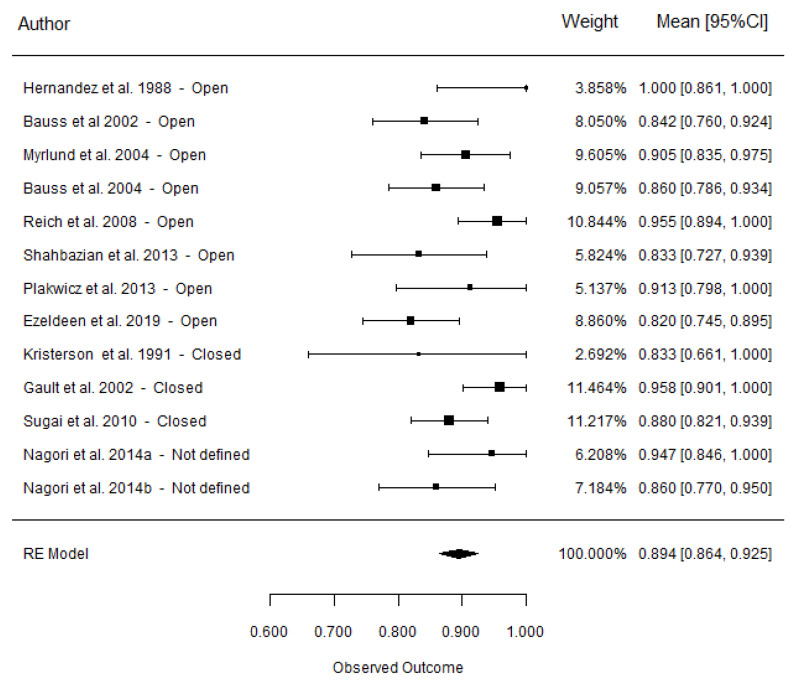
Forest plots for the overall success rate (mean [95% CI]).

The overall success rate was 89.4 ± 1.55% for 13 articles involving 712 autotransplanted teeth (Figure 4). Moderate heterogeneity was found for all studies (I^2^ = 42.5%). The success rate for the open-apex group was reported in eight, articles with a total of 545 teeth, with the average weighted success rate of 88.6 ± 2.1% and moderate heterogeneity (I^2^ = 46.5%). In the closed-apex group, the success rate was 90.9 ± 3.5%, as reported in three articles, with a total of 182 teeth (moderate heterogeneity, I^2^ = 55.0%). Meta-regression analysis of 11 studies involving 365 autotransplanted teeth resulted in a non-significant difference in success rates between the closed- and open-apex groups (*p* = 0.564) (Table 4).

The type of tooth success rate analysis was only available for the open-apex group: 87.5 ± 3.2% for premolars, and 90.6 ± 3.5% for third molars. No significant differences between premolars and third molars were observed (*p* = 0.534) (Table 4).

### 3.6. Inflammatory Root Resorption

Overall inflammatory root resorption was reported in 18 articles involving 1,233 teeth, averaging 3.8 ± 0.8%. Moderate heterogeneity was considered for all studies (I^2^ = 38.9%).

The inflammatory root resorption rate for the open-apex group reported in nine articles involving 820 teeth was 2.8 ± 0.6%. Null heterogeneity was considered for all included studies (I^2^ = 0.0%). For the closed-apex group (seven articles involving 337 teeth), the average inflammatory root resorption rate was 7.8 ± 3.1%. High heterogeneity was considered for all included studies (I^2^ = 79.5%). The meta-regression analysis of inflammatory root resorption involved 16 studies and 1157 autotransplanted teeth, and no significant differences were found between groups (*p* = 0.233).

The inflammatory root resorption rate in the open-apex group was 2.9 ± 0.7% for premolars and 2.6 ± 2.5% for third molars, without a significant difference (*p* = 0.714). In the closed-apex group, this rate was 20.7 ± 4.8% for premolars and 6.8 ± 3.9% for third molars, indicating a significant difference (*p* = 0.035) (Table 4).

### 3.7. Replacement Root Resorption

The overall replacement root resorption rate was reported in 21 articles involving 1359 teeth, with a weighted average of 4.3 ± 0.7%. Moderate heterogeneity was considered for all studies (I^2^ = 30.7%). The replacement root resorption for the open-apex group was reported in nine articles involving 919 teeth (4.0 ± 0.7%). Null heterogeneity was considered for all included studies (I^2^ = 0.0%). For the closed-apex group (seven articles involving 337 teeth), the average replacement root resorption rate was 9.0 ± 3.4%. High heterogeneity was considered for all included studies (I^2^ = 89.2%) The meta-regression analysis including 20 studies and 1340 autotransplanted teeth found no significant differences in the replacement root resorption rates (*p* = 0.471).

The replacement root resorption in the open-apex group was 4.3 ± 0.8% for premolars and 2.7 ± 1.5% for third molars, with no significant difference between the types of teeth (*p* = 0.256). In contrast, in the closed-apex group, it was 22.1 ± 0.7% for premolars and 3.8 ± 2.0% for third molars, with a significant difference (*p* = 0.003). The replacement root resorption rate in premolars was significantly higher in the closed-apex than in the open-apex group (*p* = 0.001). No statistical differences were observed in replacement root resorption for third molars between the open- and closed-apex groups (*p* = 0.660) (Table 4).

### 3.8. Root Formation

In the open-apex group, complete root formation was observed in 24.7%, incomplete in 59.4%, and arrested situation in 14.1% of the samples. 

### 3.9. Pulp Healing

In the open-apex group, pulp healing was observed in 93.5% of the cases (Table 3).

### 3.10. Pulp Obliteration 

In the open-apex group, pulp obliteration was described in 95.3% of the cases (Table 3).

## 4. Discussion

Tooth autotransplantation has shown high survival rates [42,43]. Our meta-analysis showed that tooth autotransplantation had an overall survival rate of 95.9% for both open- and closed-apex groups after a mean follow-up of 4 years. These findings are in agreement with previous systematic assessments, such as that of Machado et al., who observed survival rates of 98% and 90.5% at 1 and 5 years, respectively [44].

The present systematic review did not find statistical differences in survival, success, IER, or replacement root resorption when comparing the use of open- versus closed-apex donor teeth. Although this could indicate that the prognosis of tooth autotransplantation is not influenced by root development status, these results must be interpreted with caution because the survival rate between the groups exceeded the level of statistical significance (*p* = 0.052). On the one hand, non-significant differences when using premolars as donor teeth were observed according to the type of apex. This could be explained by the morphological similarities between anterior teeth (receptor) and single-rooted premolars (donor), resulting in a simpler and more straightforward surgical procedure, as compared to third molar transplantation. 

On the other hand, significant differences were observed in third molars, resulting in a significantly lower survival rate in the closed-apex than in the open-apex group (−3.9%). These results are in agreement with several studies that found lower survival rates in closed-apex teeth [43]. Tsukiboshi et al. stated that the younger the patient, the higher the survival rate [1]. The authors strongly believe that the differences in survival rates in wisdom teeth between both open and closed groups can mainly be attributed to the complexity of the surgical procedure. Whereas closed-apex wisdom teeth are normally related to a more complex anatomy with longer roots and a thinner PDL layer, immature wisdom teeth present shorter roots and are usually enveloped by both the dental follicle and a wider PDL, which results in a less invasive extraction. This in turn minimizes PDL damage during the procedure, as well as the risk of future complications. Another explanation could be that the regenerative capacity of PDL cells is significantly influenced by the patient’s age, as demonstrated by Zhang et al., who concluded that teeth with an open apex were less likely to fail than teeth with a closed apex [45].

Most authors agree that the predictability of this therapy depends on the survival of PDL cells during the surgical procedure [46]. Mechanical injuries to the donor tooth and prolonged extra-alveolar time during transplantation may damage the PDL, leading to progressive root resorption and failure [47,48].

In the present investigation, higher complication rates were found for IER and replacement root resorption in the closed-apex group, without reaching statistical significance. IER was 5% higher for the closed-apex group and statistically significant for premolars (20.7%) to third molars (6.8%). Furthermore, replacement root resorption was 5% higher for the closed-apex group and statistically significant for premolars (22.1%) and third molars (3.8%). These results show that ankylosis in mature teeth tends to be more than twice as common as in immature teeth. These results are in accordance with previous studies, where a clear correlation was shown between PDL surface damage and inflammatory or replacement root resorption, leading to transplant failure. Andreasen et al. found that root resorption was significantly related to increasing root development at the time of tooth transplantation [29].

Many surgical prognostic variables for successful tooth autotransplantation have been identified in recent decades, indicating that this is a highly sensitive procedure. These include age [49], plaque control [50], smoking habits [50], donor tooth anatomy [17,18,51], periodontal condition [18,19,49], root development status [52], eruptive stage [12,17,52,53], extra-alveolar time [1,11,46], recipient site integrity [19,52], and difficulty of the extraction and splinting method [18,42]. This has resulted in a gradual evolution of the surgical protocol [54,55]. 

Antiseptics play an important role after any surgical procedure due to bactericidal and bacteriostatic properties, reducing the risk of post-operative infection. The use of chlorhexidine could be potentially beneficial after a tooth autotransplantation, helping to control dental plaque, which could impact periodontal healing [56].

Advances in the field of tooth autotransplantation in the last decade include the use of digital planning, computer-aided rapid prototyping (CARP) models, and fully guided surgical protocols [55]. In 2001, Lee et al. first proposed the use of CARP models during tooth autotransplantation [57] to reduce the extraoral time of the donor tooth, mechanical damage to its PDL, and bone socket remodeling, thus relieving the patient’s post-operative pain. These advancements allowed for reduced manipulation of the donor tooth, minimizing the extra-alveolar time and thus increasing the chances of long-term success [54].

No significant differences were found in success rates between mature and immature teeth; however, this might be related to the high variability of success criteria among the studies. Most studies assessing closed-apex teeth described only survival rates and not success rates [10,12,22,27,29,30,31,34,35,36,39]. This lack of success assessment in a number of studies including closed-apex teeth could be explained by the traditional inclusion of pulp vitality signs as part of the success criterion definition, as only developing donor teeth were considered [9]. 

Stem cells derived from apical papilla (SCAP) found in the immature root apex [58] are responsible for tooth vitality after transplantation. Unlike fully developed teeth, the pulp of immature teeth can heal/repair after transplantation [29]. The most significant predictors of pulpal healing appear to be the width and length of the root canal, as well as the duration and type of extra-alveolar storage [59]. 

According to some studies, tooth autotransplantation should ideally be performed when root formation has achieved 1/2 or 2/3 of the expected complete development [12,27,30]. This condition corresponds with a radiographically open apex, which allows for revascularization of the pulp and continued root growth [60]. Hence, root canal treatment (RCT) is not indicated after immature tooth transplantation, in contrast to fully developed transplanted teeth, in which RCT is indicated within the first 4 weeks after transplantation, as pulp regeneration is not expected [29].

Andreasen et al. reported a completed development of the root in 21% of open-apex teeth cases, incomplete development in 65%, and arrested development in 14% [10]. These results are in accordance with our review, in which 24.7% of cases showed completed root development; 59.4%, incomplete; and 14.1%, arrested. 

This review is not without limitations. No randomized clinical trials fulfilled the inclusion criteria, and some included studies presented insufficient data when reporting some clinical parameters, such as success rate [10,12,22,27,29,30,31,34,35,36,39], replacement root resorption [12,24,29,35,41], inflammatory root resorption [11,12,24,29,32,35,36,39], pulp healing [10,24,25,26,29], pulp obliteration [10,12,22,23,24,26,28,29,33], and root formation [10,11,12,22,26,28,32]. Additionally, moderate and high heterogeneity was found when results were explained, risking ambiguity in the described information, lack of standardization of the success-rate criteria, or misidentification of the follow-up duration. 

The results from this meta-analysis represent a limited level of evidence and therefore must be interpreted with caution. Additional studies with strong levels of evidence involving randomized controlled clinical trials, prospective and well-designed clinical studies, longer follow-ups, and a general agreement on the success rate criteria are necessary in the future to examine and to confirm the outcomes obtained from the present systematic review assessing the prognosis of tooth autotransplantation. 

In conclusion, tooth autotransplantation could be considered a viable treatment option with high survival (95.9 ± 0.8%) and success rates (89.4 ± 1.55%) after a mean follow-up of 4 years. Open-apex donor teeth may be preferable, implying a 3.9% higher survival rate and lower complication rates compared to closed-apex teeth. Ankylosis in mature teeth tends to be more than twice as common as in immature teeth. Non-significant differences were observed when using premolars as a donor teeth according to the type of apex, whereas significant differences were observed in third molars, resulting in a significantly lower survival rate in the closed-apex than in the open-apex group.

## Figures and Tables

**Table 1:** Characteristics and study design of studies included in the analysis.

Author	Study Design	Follow-Up (Years)	Nº Patients	Nº Teeth	Age Range	Donor Tooth Type		Splinting Procedure		Splinting Duration		Occlusion/Infraocclusion	3D Replica
						Maxilla	Mandible	Suture	Wire	Suture	Wire		
**OPEN APEX**													
**Kristerson et al. 1985** [27].	PCS	3–18Y (6.3Y)	NR	82	10.0–19	PM (84)		Yes (19)	Yes (63)	1 W	0	I/O	NR
**Hernandez et al. 1988** [28].	PCS	3Y	10	10	13–19	TM (10)		Yes (10)	0	2 W	0	I	Yes
**Andreasen II et al. 1990** [12].	PCS	1–13Y	NR	317	7–35.0	PM (317)		Yes	Yes	1 W	0	NR	NR
**Andreasen III et al. 1990** [10].	PCS	1–13Y	NR	317	7–35.0	PM (317)		Yes	Yes	1 W	0	NR	NR
**Andreasen IV et al. 1990** [29].	PCS	1–13Y	NR	317	7–35.0	PM (317)		Yes	Yes	1 W	0	NR	NR
**Paulsen et al. 1995** [30] **/1998** [31].	PCS	6–18Y	NR	118	NR	PM (104)	PM (14)	0	0	0	0	NR	NR
**Bauss et al. 2002** [32].	PCS	1.0–6.1Y (3.4Y)	72	76	16.3–20.3	TM (40)	TM (36)	Yes (42)	Yes (34)	1 W	4 W	I	NR
**Myrlund et al. 2004** [24].	PCS	4 Y	54	68	6.5–20	PM (68)		0	0	0	0	I	NR
**Bauss et al. 2004** [25].	PCS	1–6.3Y (3.4Y)	79	85	16.1–20.3	TM (85)		Yes	Yes	1 W	4 W	I	NR
**Reich et al. 2008** [33].	PCS	6 m–4Y (1.7Y)	32	44	11.0–25	TM (44)		Yes (44)	0	2 W	0	I	NR
**Yan et al. 2010** [22].	PCS	1–11Y (5.2Y)	NR	16	16–39	TM (16)		Yes (16)	Yes (11)	1 W	1 W	NR	NR
**Shahbazian et al. 2013** [23].	PCS	1Y	40	48	9.0–18	M (4) PM (44)		0	Yes (48)	0	Few W	I	Yes
**Plakwicz et al. 2013** [11].	PCS	6–78m (2.11Y)	19	23	9.1–17	2PM (17)	2PM (6)	Yes (23)	0	2 W	0	I	NR
**Ezeldeen et al. 2019** [26].	PCS	1.1–10.4Y (4.5Y)	88	100	8.0–13	PM (100)		Yes	Yes	0	0	I	Yes
**CLOSED APEX**													
**Azaz et al. 1978** [36].	PCS	2–7Y	31	37	13–36.0	Cs (37)	0	0	Yes (37)	0	10 weeks	NR	NR
**Kristerson et al. 1985** [27].	PCS	3–18Y (6.3Y)	NR	18	15–58.0	PM (18)		0	Yes (18)	0	0	O	NR
**Andreasen II et al. 1990** [12].	PCS	1–13Y	NR	53	7–35.0	PM (53)		Yes	Yes	1 W	0	NR	NR
**Andreasen III et al. 1990** [10].	PCS	1–13Y	NR	53	7–35.0	PM (53)		Yes	Yes	1 W	0	NR	NR
**Andreasen IV et al. 1990** [29].	PCS	1-13Y	NR	53	7–35.0	PM (53)		Yes	Yes	1 W	0	NR	NR
**Kristerson et al.1991** [37].	PCS	1.5–6Y	18	18	24–58	TM (18)		Yes (18)	Yes (18)	1 W	2-3 W	NR	NR
**Gault et al. 2002** [38].	PCS	2–7Y (5Y)	43	47	33–73	M (43), PM (2) and C (2)		Yes (47)	0	2 W	0	I	NR
**Mejàre et al. 2004** [39].	PCS	1–10Y(4Y)	47	47	21–66	TM (47)		Yes (47)	Yes (10)	10 days	10 days	I	NR
**Arikan et al. 2008** [35].	PCS	2–8Y(5.87Y)	30	32	25–55	C (31)	0	0	Yes (32)	0	4 W	NR	NR
**Yan et al. 2010** [22].	PCS	1–11Y (5.2Y)	NR	19	16–39	TM (19)		Yes (19)	Yes (13)	1 W	1 W	NR	NR
**Sugai et al. 2010** [18].	PCS	1–5.9Y (3.40Y)	109	117	11–75.0	M (40) PM (10) I (1)	M (10) PM (16)	Yes (117)	Yes (117)	1 W	3 W	I	NR
**Yu et al. 2017** [34].	PCS	7–13Y (9.9Y)	60	65	19–55	TM (65)		Yes (65)	0	2-3 W	0	NR	NR
**NOT DEFINED**													
**Nagori et al. 2014a** [40].	PCS	1.4Y	19	19	16–25	TM (19)		Yes (12)	Yes (7)	1 W	2 W	I	NR
**Nagori et al. 2014b** [41].	PCS	1.25–2Y (1.65Y)	53	57	15–25	TM (17)	TM (40)	Yes (57)	Yes (12)	1 W	2 W	I	NR

Abbreviations: PCS, prospective case series; Y, years; PM, premolars; TM, third molars, C, canines; M, molars; W, week; O, occlusion; I, infraocclusion; NR, not reported.

**Table 2:** Assessment of the included prospective case series studies using ROBINS-I.

Study	*Pre-Intervention*		At Intervention	*Post-Intervention*				Overall Risk of Bias
	Bias Due to Confounding	Bias in the Selection of Participants in the Study	Bias in Classification of Intervention	Bias Due to Deviation from Intended Interventions	Bias Due to Missing Data	Bias in Measurement of Outcomes	Bias in Selection of the Reported Result	
Azaz et al. 1978 [36].	Moderate	Low	Low	Low	Low	Moderate	Moderate	Moderate
Kristerson et al. 1985 [27].	Low	Low	Low	Low	Low	Moderate	Low	Low
Hernandez et al. 1988 [28].	Moderate	Low	Low	Low	Low	Moderate	Low	Moderate
Andreasen II et al. 1990 [12].	Moderate	Low	Low	Low	Low	Moderate	Moderate	Moderate
Andreasen III et al. 1990 [10].	Moderate	Low	Low	Low	Low	Moderate	Moderate	Moderate
Andreasen IV et al. 1990 [29].	Moderate	Low	Low	Low	Low	Moderate	Moderate	Moderate
Kristernson et al. 1991 [37].	Low	Low	Low	Low	Low	Moderate	Low	Low
Paulsen et al. 1995 [30].	Low	Low	Low	Low	Low	Moderate	Low	Low
Paulsen et al. 1998 [31].	Low	Low	Low	Low	Low	Moderate	Low	Low
Bauss et al. 2002 [32].	Low	Low	Low	Low	Low	Moderate	Low	Low
Gault et al. 2002 [38].	Low	Low	Low	Low	Low	Moderate	Low	Low
Myrlund et al. 2004 [24].	Moderate	Low	Low	Low	Low	Moderate	Moderate	Moderate
Bauss et al. 2004 [25].	Low	Low	Low	Low	Low	Moderate	Low	Low
Mejàre et al. 2004 [39].	Moderate	Low	Low	Low	Low	Moderate	Moderate	Moderate
Reich et al. 2008 [33].	Low	Low	Low	Low	Low	Moderate	Moderate	Moderate
Arikan et al. 2008 [35].	Moderate	Low	Low	Low	Low	Moderate	Moderate	Moderate
Yan et al. 2010 [22].	Moderate	Low	Low	Low	Low	Moderate	Low	Moderate
Sugai et al. 2010 [18].	Low	Low	Low	Low	Low	Moderate	Low	Low
Shahbazian et al. 2013 [23].	Moderate	Low	Low	Low	Low	Moderate	Low	Moderate
Plakwicz et al. 2013 [11].	Moderate	Low	Low	Low	Low	Moderate	Low	Moderate
Nagori et al. 2014 [40].	Moderate	Low	Critical	Low	Low	Moderate	Moderate	Moderate
Nagori et al. 2014 [41].	Moderate	Low	Critical	Low	Low	Moderate	Moderate	Moderate
Yu et al. 2017 [34].	Low	Low	Low	Low	Low	Moderate	Low	Low
Ezeldeen et al. 2019 [26].	Low	Low	Low	Low	Low	Moderate	Moderate	Moderate

**Table 3:** Outcomes for the survival, success, root resorption, pulp condition, and root formation.

Author	Survival (%)	Success (%)	Root Resorption		Pulp Condition		Root Formation		
			Inflammatory Root Resorption (%)	Replacement Root Resorption (%)	Pulp Healing (%)	Pulp Obliteration (%)	Complete (%)	Incomplete (%)	Arrested (%)
**OPEN APEX**									
Kristerson et al. 1985 [27].	96.34	NR	2.43	7.31	90.25	100	57.31	42.69	0
Hernandez et al. 1988 [28].	100	100	0	0	100	NR	NR	NR	NR
Andreasen II et al. 1990 [12].	95	NR	NR	NR	95.9	NR	NR	NR	NR
Andreasen III et al. 1990 [10].	NR	NR	2.52	3.78	NR	NR	NR	NR	NR
Andreasen IV et al. 1990 [29].	NR	NR	NR	NR	NR	NR	21	65	14
Paulsen et al. 1995 [30] /1998 [31].	93.3	NR	3.38	4.23	86.45	100	26	55	19
Bauss et al. 2002 [32].	100	84.2	NR	5.3	90.8	90.8	NR	NR	NR
Myrlund et al. 2004 [24].	98.6	90.5	NR	NR	NR	NR	19.1	54.4	26.5
Bauss et al. 2004 [25].	100	86	8.23	4.7	NR	87.05	NR	NR	19
Reich et al. 2008 [33].	95.5	95.5	0	0	100	NR	0	44	0
Yan et al. 2010 [22].	100	NR	0	0	87.5	NR	NR	NR	NR
Shahbazian et al. 2013 [23].	95.83	83.33	4.16	10.41	95.84	NR	NR	60.41	NR
Plakwicz et al. 2013 [11].	100	91.3	NR	4.34	100	100	NR	NR	NR
Ezeldeen et al. 2019 [26].	88	82	5	5	NR	NR	NR	NR	NR
**CLOSED APEX**									
Azaz et al. 1978 [36].	89.18	NR	NR	32.43					
Kristerson et al. 1985 [27].	77.77	NR	27.77	33.33					
Andreasen II et al. 1990 [12].	98	NR	NR	NR					
Andreasen III et al. 1990 [10].	NR	NR	18.86	16.98					
Andreasen IV et al. 1990 [29].	NR	NR	NR	NR					
Kristerson et al. 1991 [37].	88.88	83.33	0	5.55					
Gault et al. 2002 [38].	95.75	95.75	0	0					
Mejàre et al. 2004 [39].	81.4	NR	NR	2.12					
Arikan et al. 2008 [35].	93.5	NR	NR	NR					
Yan et al. 2010 [22].	89.47	NR	10.52	0					
Sugai et al. 2010 [18].	96	88	4.27	4.27					
Yu et al. 2017 [34].	90.8	NR	10.8	9.2					
**NOT DEFINED**									
Nagori et al. 2014a [40].	94.73	94.73	0	0	NR	NR	NR	NR	NR
Nagori et al. 2014b [41].	98.24	86	10,28	NR	NR	NR	21.05	78.95	NR

Abbreviation: NR, not reported.

**Table 4:** Outcomes comparing the survival, success, and root resorption rates depending to the apex condition and tooth type.

	Survival (%)	Success (%)	Root Resorption	
			Inflammatory Root Resorption (%)	Replacement Root Resorption (%)
**Overall**	95.9 ± 0.8	89.4 ± 1.55	3.8 ± 0.8	4.3 ± 0.7
**Open apex**	96.9 ± 1.0	88.6 ± 2.1	2.8 ± 0.6	4.0 ± 0.7
**Closed apex**	93.0 ± 1.7	90.9 ± 3.5	7.8 ± 3.1	9.0 ± 3.4
***p*-value**	*p* = 0.052	*p* = 0.564	*p* = 0.233	*p* = 0.471
**Canines**				
**Open apex**	(-)	(-)	(-)	(-)
**Closed apex**	91.6 ± 3.3	(-)	(-)	(-)
***p*-value**	(-)	(-)	(-)	(-)
**Premolars**				
**Open apex**	95.5 ± 1.2	87.5 ± 3.2	2.9 ± 0.7	4.3 ± 0.8
**Closed apex**	90.2 ± 9.8	(-)	20.7 ± 4.8	22.1 ± 0.7
***p*-value**	*p* = 0.797	(-)	(-)	*p* = 0.001
**Third molars**				
**Open apex**	99.7 ± 0.8	90.6 ± 3.5	2.6 ± 2.5	2.7 ± 1.5
**Closed apex**	88.4 ± 2.6	(-)	6.8 ± 3.9	3.8 ± 2.0
***p*-value**	*p* = 0.001	(-)	(-)	*p* = 0.660
**Open apex**				
**Premolars**	95.5 ± 1.2	87.5 ± 3.2	2.9 ± 0.7	4.3 ± 0.8
**Third Molars**	99.7 ± 0.8	90.6 ± 3.5	2.6 ± 2.5	2.7 ± 1.5
***p*-value**	*p* = 0.008	*p* = 0.534	*p* = 0.714	*p* = 0.256
**Closed apex**				
**Premolars**	90.2 ± 9.8	(-)	20.7 ± 4.8	22.1 ± 0.7
**Third Molars**	88.4 ± 2.6	(-)	6.8 ± 3.9	3.8 ± 2.0
***p*-value**	*p* = 0.046	(-)	*p* = 0.035	*p* = 0.003
